# Recommendations for increasing yield of the edible *Pinus pinea* L. pine nuts

**DOI:** 10.1371/journal.pone.0300008

**Published:** 2024-03-05

**Authors:** Verónica Loewe-Muñoz, Claudia Delard, Rodrigo del Río, Mónica Balzarini

**Affiliations:** 1 Chilean Forest Institute (INFOR), Santiago, Chile; 2 Centro Nacional de Excelencia para la Industria de la Madera (CENAMAD), Pontificia Universidad Católica de Chile, Santiago, Chile; 3 CONICET UFYMA Biometry Unit, Universidad Nacional de Córdoba, Córdoba, Argentina; Technical University in Zvolen, SLOVAKIA

## Abstract

In *Pinus pinea*, cone to pine nut yield (total pine nut weight expressed as percentage of cone weight), an important crop trait, is decreasing worldwide. This phenomenon is of great concern, since the nuts of this species are highly demanded. Cone weight, seed and pine nut morphometry, and pine nut yield were monitored in a non-native area in Chile for 10 years. For this purpose, 560 cones, and the seeds and pine nuts contained in them, were counted, measured and weighed in a multi-environment study involving seven plantations. Seed and pine nut damage was evaluated. Two contrasting categories of cone weight (heavy/light) were defined. Cone to pine nut yield (PY) and other traits were calculated and compared between categories using a mixed linear model. Regression trees were used to explain PY variability. Cone weight was higher than in the species’ native range (474 g vs 300 g on average). Pine nut number per cone and PY were significantly higher in the heavy cone category than in the light cone category (125 vs 89 units, and 4.05 vs 3.62%, respectively), The percentage of damaged seeds was lower in heavy than in light cones (9.0% vs 15.9%). Thus, PY depended on seed and pine nut morphometry as well as on seed health. Management practices, such as fertilization and irrigation, could be used to boost production of heavy cones and consequently increase PY.

## Introduction

*Pinus pinea* L., commonly known as stone pine, is highly appreciated for its pine nuts; indeed, nut chemical composition includes high quality fats, proteins, vitamins, minerals and bioactive compounds [1]. The species, one of the most important nut species in the world, is harvested from native forests or plantations in Spain, Portugal, Italy, Turkey and Tunisia [2]. In has been consumed since ancient times [3]. Genetic material selection has been initiated and horticultural management techniques are being developed [4] with the aim to domesticate the species as an agronomic nut crop.

As far back as 2,800 years ago, stone pine was planted outside its native distribution range [5]. Due to the pine nut market opportunities, stone pine cropping has expanded to non-native countries, like Argentina [6], Australia [7], New Zealand [8] and Chile [9]. Significant efforts are being made to maximize pine nut production [10]. This is particularly important because only a very small fraction of cone weight corresponds to pine nuts [11]. Pine nut production, which exhibits a high inter-annual variability [12], is related to the number of cones and to the morphometric characters that define cone to pine nut yield (PY).

In the last decades, a severe reduction of cone to seed yield (from 17% to 5%) and PY (from 4% to 2%, or even less) has been reported in Europe [13], with apparently healthy cones containing up to 50% of empty seeds. This fact is relevant, since companies buy cones by weight instead of pine nut content [14]. Thus, the importance of PY monitoring has increased due to the growing presence of empty and damaged seeds [15], which was attributed to biotic (*Leptoglossus occidentalis*, [16, 17]) and abiotic damage (droughts, [13, 17]). The insect *L*. *occidentalis* was detected in Chile in 2017 [18, 19] and in Argentina in 2019 [20]. Even though cone weight was found to be correlated to pine nut number and weight [21, 22], a non-significant correlation was found between cone weight and PY in Chile [23].

The production of bigger cones has been related to an improved fruit quality [21]. Therefore, management practices such as fertilization and irrigation could be implemented to boost production of heavy cones, thereby improving PY. The objective of this study was to compare the number of damaged seeds and PY among different stone pine cone weight categories across a wide range of spatial-temporal variability in Chile. Our working hypothesis was that heavy cones would have a higher PY and a lower percentage of damaged seeds than light cones. Knowledge of PY is necessary to boost stone pine cropping in the local industry. Comparing harvest traits with values reported for the species in other parts of the world is of interest to the international industry and associated organizations.

## Material and methods

### Material

Cones were sampled in seven adult stone pine plantations located in an area extending between Valparaiso and Araucanía regions in Chile. The location of the plantations is presented in Table 1, along with general climatic characteristics (average climatic values during the 2010–2020 period). Table 2 presents a description of stands, including dendrometric variables.

**Table 1:** Characterization of the studied stone pine plantations.

Plantation	Geographical Location			Annual average temperature (°C)	Autumn maximum temperature† (°C)	Annual rainfall (mm)
	Latitude	Longitude	Altitude (m a.s.l.)			
Santo Domingo	33°38’ S	71° 37’ W	31	12.4	17.4	304
Rosario	34°20’ S	70°51’ W	352	13.6	18.6	300
Cáhuil	34°29’ S	72° 0’ W	116	13.2	18.7	382
Quilvo	34°55’ S	71° 7’ W	330	14.2	18.0	459*
Toconey	35°24’ S	72° 3’ W	56	14.2	20.1	570
Mulchén	37°39’ S	72°15’ W	408	13.2	17.5	1,150
Antiquina	38°04’ S	73°23’ W	100	11.5	15.2	815

* with irrigation in spring and summer.

† Autumn (March 21^st^ to June 20^th^) maximum temperature was found to be a significant variable for seed number cone^-1^ [9].

Climatic data were obtained from the Chilean National Environmental Information System (www.dga.cl; www.inia.cl).

**Table 2:** Characterization of stone pine plantations (2020).

Plantation	Age (years)	Tree size		Plantation area (m^-2^)	Spacing (m × m)
		DBH (cm)	Height (m)		
Santo Domingo	33	28.5	15.2	1,500	6**×**6
Rosario	25	51.1	12.8	6,000	6**×**6
Cáhuil	37	23.2	6.4	12,000	7**×**7
Quilvo	21	52.3	11.6	180	4**×**4
Toconey	27	34.9	14.2	21,000	7**×**7
Mulchén	51	50.1	14.7	500	5**×**5
Antiquina	23	31.7	13.8	400	2**×**3

All plantations were longitudinally sampled to obtain 10 cones per stand in winter during the 2010–2020 period, except for 2016. A hierarchical random sampling was used to select 10 trees and one healthy 3-year-old cone per tree. Therefore, 10 cones were randomly harvested per plantation each year. In some years the number of collected cones was lower than 10 due to harvesting complexities. Fresh weight of cones was immediately recorded, as previously indicated [24, 25].

Each year, the harvested cones were processed at INFOR’s laboratory to extract seeds (in-shell pine nuts) and pine nuts (kernels); in total, 560 cones were harvested throughout the study period. Seed number per cone was counted and seed and pine nuts were weighed and measured in the laboratory using the procedures indicted in Table 3. Cone to seed yield, seed to pine nut and PY were calculated using the formulas presented in Table 3. Empty and damaged seeds were also quantified to monitor cone health. We measured all in-shell seeds and shelled pine nuts per cone; for the 2010–2015 period, we only have aggregated data. Between 2017 and 2020, we counted and weighed all the in-shell and shelled pine nuts per cone, and measured a random sample of 20 in-shell and shelled pine nuts per cone for all traits [26].

**Table 3:** Stone pine fruit traits.

Traits	Abbreviation	Unit	Measurement procedures
Cone weight	CW	g	3-year-old cones were weighed in a Mettler (Toledo, Spain) AJ150 †
Cone length	CL	mm	Measured with a digital caliper
Cone diameter	CD	mm	Measured with a digital caliper in the largest section
Seeds per cone	SN	#	All seeds were extracted from each cone and counted
Seed weight	SW	g	Each seed per cone was weighed in a Mettler (Toledo, Spain) AJ150 ††
Seed length	SL	mm	Measured with a digital caliper ††
Seed diameter	SD	mm	Measured with a digital caliper in the largest section ††
Seed yield	SY	%	*SY* = ((*SN*×*SW*)/*CW*)×100
Seed to pine nut yield	SPY	%	*SPY* = ((*PN*×*PW*)/*total seed weight per cone*)×100
Pine nuts per cone	PN	#	All healthy pine nuts from each cone were counted
Pine nut weight	PW	g	Each pine nut was weighed in a Mettler (Toledo, Spain) AJ150 †††
Pine nut length	PL	mm	Measured with a digital caliper ††††
Pine nut diameter	PD	mm	Measured with a digital caliper in the largest section ††††
Pine nut yield	PY	%	*PY* = ((*PN*×*PW*)/*CW*)×100
Empty/Damaged seeds	DS	%	*DS* = ((*SN*×*PN*)/*SN*×100

† Fresh weight at harvest

†† For all seeds per cone for the 2010–2015 period, and for 20 seeds per cone for the 2017–2020 period.

††† Pine nuts were previously dried to 6% of humidity at 40°C in a Red Line Binder oven (Tuttlingen, Germany)

†††† For all healthy pine nuts per cone for the 2010–2015 period, and in 20 healthy pine nuts per cone for the 2017–2020 period.

### Statistical analyses

First, cone weight categories were defined as the extreme tertiles of cone weight probabilistic distribution using all harvested cones across plantations and years (n = 560). Then, mean differences in morphometric traits, percentage of damaged seeds and PY were compared between cone weight categories (light and heavy cones) using a multi-environment ANOVA mixed model (α = 0.05), including cone weight category and plantation as fixed effects, and year as well as the corresponding interactions as random effects [27]. The ANOVA mixed model was fitted with homogeneous and heterogeneous variances for each cone weight category. The Akaike information criteria (AIC) was used to select the best model (the heteroscedastic model).

In addition, regression tree (RT) analysis [28] was used to explain PY variability in traits of cones, seeds and pine nuts. Box plots were used to describe the distribution of the main variables affecting PY for each plantation, according to the literature [27]. Statistical analyses were performed using the software InfoStat [29] and its interface with R (www.r-project.org).

## Results

Across plantations and years, cone weight was on average 470 g, with an average PY of 3.91%. No significant interaction between cone weight category and plantation (p>0.05) was found for any trait. Weight of light and heavy cones was below 393 g and above 503 g, respectively. Compared with the light cone category, heavy cones had a 40.0% higher number of seeds per cone (125 vs 89 seeds per cone, respectively), 26.3% higher seed weight, larger seeds (13.3% and 9.5% greater length and width, respectively) and 5.8% higher cone to seed yield. The heavy cone weight category also had a 48.4% higher number of pine nuts per cone (112.9 vs 76.1 pine nuts per cone, respectively), 22.2% higher pine nut weight, 13.0% longer pine nuts, 4.1% wider pine nuts, 11.9% higher PY (4.05 vs 3.62%) and a lower number of damaged seeds (9.0% vs 15.9% damaged seeds) (Table 4).

**Table 4:** Stone pine fruit traits by cone weight category.

Variable	Unit	Heavy cones (> 503 g)	Light cones (< 393 g)	Overall mean
CW	g	594.9±5.7 a	338.0±4.7 b	473.6±25.8
CL	mm	152.8±19.5 a	131.3±20.3 a	182.9±34.2
CD	mm	116.2±16.0 a	103.3±17.2 a	144.9±27.3
SN	#	124.8±3.4 a	89.1±3.6 b	111.4±3.3
SW	#	0.96±0.02 a	0.76±0.02 b	0.85±0.06
SL	mm	18.8±0.5 a	16.6±0.6 b	17.9±0.6
SD	mm	9.2±0.3 a	8.4±0.3 b	8.8±0.2
SY	%	19.8±0.6 a	18.7±0.6 b	19.7±0.6
PN	#	112.9±3.4 a	76.1±3.7 b	97.5±4.2
PW	g	0.22±0.01 a	0.18±0.01 b	0.19±0.02
PL	mm	13.8±0.4 a	12.2±0.5 b	13.3±0.4
PD	mm	5.0±0.3 a	4.8±0.3 b	5.0±0.1
SPY	%	20.9±0.8 a	20.3±0.9 a	20.3±0.9
PY	%	4.05±0.17 a	3.62±0.18 b	3.91±0.16
DS	%	9.0±1.6 b	15.9±2.0 a	12.8±2.3

CW: cone weight, CL: cone length, CD: cone diameter, SN: seeds per cone, SW: seed weight, SL: seed length, SD: seed diameter, SY: seed yield, PN: pine nuts per cone, PW: pine nut weight, PL: pine nut length, PD: pine nut diameter, SPY: seed to pine nut yield, PY: pine nut yield, DS: empty/damaged seeds.

Mean values ± standard error.

**Fig 1:**
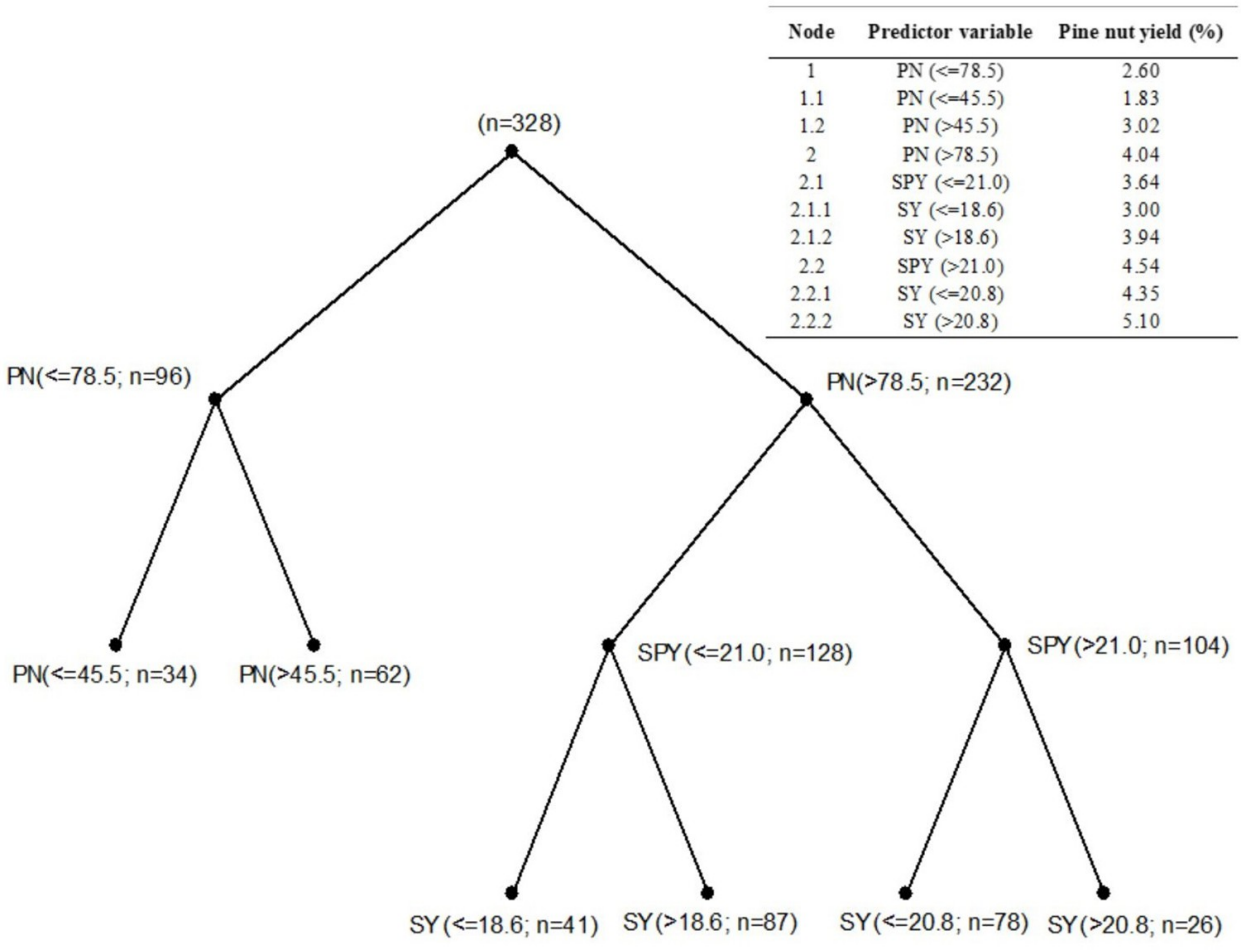
Fruit variables that best explained cone to pine nut yield in Chile (light and heavy cone weight categories). PY data were first split into two subsets based on the predictor variable (PN) and its threshold (78.5). Each subset, or node, was then analyzed independently using the same procedure. Variables forming top nodes are the most important to explain PY. Average PY values for each node are reported in the embedded table. PN: pine nuts per cone; SPY: seed to pine nut yield; SY: cone to seed yield.

Regression tree analysis showed that PY was influenced by pine nut number per cone. PY decreased by 35.6% when cones contained fewer than 78.5 units; PY averages were 4.04% and 2.60% below and above that threshold, respectively. Cones with fewer than 45.5 units showed a further yield reduction of 39.4%. Cones containing 78.5 pine nuts or more, and that had a seed to pine nut yield above 21.0% had a higher PY (4.54% vs 3.64%) than cones that had a seed to pine nut yield below 21.0%. Moreover, in cones containing 78.5 pine nuts or more, and seed to pine nut yield above 21.0%, PY further increased when cone to seed yield was above 20.8% (5.10% vs 4.35%) (Fig 1).

**Fig 2:**
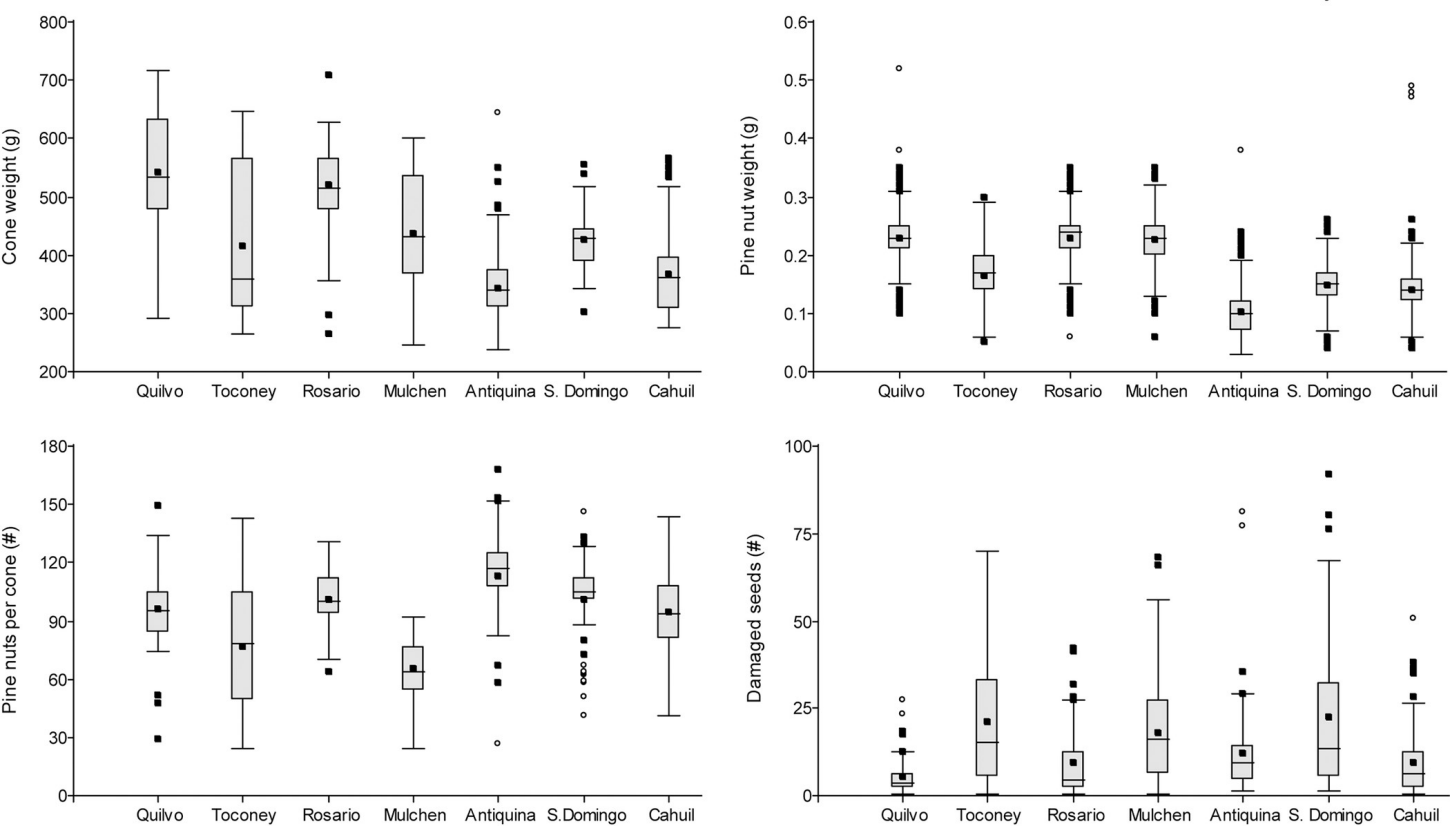
Distribution of cone and pine nut weight, and number of healthy pine nuts and damaged seeds per cone for each sample site in the 2017–2020 period.

The distribution of variables related to PY for all sites is presented at the seed or pine nut level in Fig 2 for the 2017–2020 period.

## Discussion

Size is an important quality attribute in most fruit crops [30]. In stone pine, the relationship between cone quality and cone weight was analyzed; size and cone weight were found to be statistically correlated and cone weight was also correlated with seed and pine nut weight [23, 31]. Cone weight has shown to be affected by climatic conditions in the spring of the last year of cone maturation, especially by rainfall [32, 33], which partially explains the high inter-annual variability previously reported in the species’ native habitat [34]. In Chile [9], annual rainfall [35] and average temperature were also positively correlated with cone weight.

There is a known dependence of PY on number of pine nuts inside cones [20]; the relationship of PY and cone weight is becoming a cause of concern due to the effect of pests and diseases on seeds. In this study, cone weight was on average 470 g, which is higher than in the species’ native range [34, 36–40]. In the 2010–2020 period in Chile, seed to pine nut yield was on average 20.3% across sites and years; these results are in line with values reported for the species’ native range [36, 41–43]. Similarly, average PY value (3.9%) is also within the range of historical values reported for Italy (3.6% [40, 44] and Spain (2.7–4.4% [45]). However, the average PY value is higher than values reported for Europe after the arrival of *L*. *occidentalis*, which caused a drastic decrease in Spain (1.1–2.1% [31, 45]) and Portugal (1.7% [46]; 3.0% [44]). In Chile, a trend towards PY decrease was also reported [23, 47].

Regarding cone weight, our results showed that the PY value of the heavy cone category was 11.9% higher than that of the light category across sites and years, in agreement with [36]. This result disagrees with findings that showed no effect of cone weight on PY in the species’ native habitat [32]. This PY increase in the heavy cone category is explained by the increase in the number of healthy pine nuts per cone (48.4%) and pine nut weight (22.2%), the most influential variables in determining PY [23]. The measured values of pine nuts per cone (cone filling) are higher than those reported for Turkey [48], Italy [49] and Portugal [22], leading to an increased production. The fact that PY is higher in heavier fruits has been reported for other crops [50–52], with composed fruits, formed from one flower and containing several seeds, as occurs in stone pine.

In agreement with previous findings [53], the number of damaged seeds was significantly lower in the heavy cone than in the light cone weight category, which favors PY. The average proportion of damaged seeds (12.8%) is similar to that reported for Croatia [54] and lower than those reported for Tunisia (19.3% [55]), Spain (50% [56], 60% [57]) and Lebanon (60% [58]). However, the average number of damaged seeds was twice as high as the value reported for Chile in 2018 [59]. This difference could be due to damage by *L*. *occidentalis* and/or to the severe uninterrupted mega-drought that has affected the country since 2010, with rainfall deficits of up to 40% [60]. The distribution of PY-related variables for Quilvo, Cáhuil and Rosario–geographically close locations with a similar rainfall regime–is not enough to attribute the observed differences to the irrigation provided in Quilvo.

Cone weight is highly variable within plantations; therefore, the differences in PY between the heaviest and the lightest cones are not trivial. However, our results indicate that cone weight may be used as an indicator of stone pine cone quality, in agreement with [36, 61]. In fact, our study showed that bigger cones contain a higher number of seeds (unshelled pine nuts), higher yield and bigger pine nuts (shelled white pine nuts, the edible component), as previously reported [36].

This longitudinal multi-environment study showed the dependence of PY on cone weight; hence, management practices, such as fertilization and irrigation, could be used to boost production of heavy cones. In fact, previous studies reported that fertilization increased PY [61] and cone weight [62, 63]. The benefits of fertilization with both micronutrients [64, 65] and macronutrients [21, 38] have been studied in adult stone pine trees. On the other hand, irrigation has also been found to improve cone weight in stone pine [61, 66, 67]. Therefore, further studies targeting these and other management practices to increase cone weight should explore tools to boost PY, with the consequent economic benefits.

## Conclusions

In Chile, stone pine cones were found to be heavier than in the species’ native habitat. PY depends on seed and pine nut morphometry and seed health. Heavy cones contained a higher (48.4%) number of healthy pine nuts, and higher cone to seed and seed to pine nut yields, and consequently 11.9% higher PY than light cones. Management practices that increase cone weight, such as fertilization and irrigation, are recommended to increase PY.
